# Interleukin-15 antagonizes muscle protein waste in tumour-bearing rats

**DOI:** 10.1054/bjoc.2000.1299

**Published:** 2000-07-24

**Authors:** N Carbó, J López-Soriano, P Costelli, S Busquets, B Alvarez, F M Baccino, L S Quinn, F J López-Soriano, J M Argilés

**Affiliations:** Departament de Bioquímica i Biologia Molecular, Facultat de Biologia, Universitat de Barcelona, Diagonal 645, 08071-Barcelona, Spain; Dipartimento di Medicina ed Oncologia Sperimentale, Università di Torino, Torino, Italy; Centro CNR di Immunogenetica ed Oncologia Sperimentale, Torino, Italy; Geriatric Research, Education and Clinical Center, VA Puget Sound Health Care System, American Lake Division, Tacoma, Washington, 98493, USA

**Keywords:** IL-15, cancer cachexia, protein turnover, skeletal muscle, proteasome, ubiquitin

## Abstract

Tissue protein hypercatabolism (TPH) is an important feature in cancer cachexia, particularly with regard to the skeletal muscle. The Yoshida AH-130 rat ascites hepatoma is a model system for studying the mechanisms involved in the processes that lead to tissue depletion, since it induces in the host a rapid and progressive muscle wasting, primarily due to TPH. The present study was aimed at investigating if IL-15, which is known to favour muscle fibre hypertrophy, could antagonize the enhanced muscle protein breakdown in this cancer cachexia model. Indeed, IL-15 treatment partly inhibited skeletal muscle wasting in AH-130-bearing rats by decreasing (8-fold) protein degradative rates (as measured by^14^C-bicarbonate pre-loading of muscle proteins) to values even lower than those observed in non-tumour-bearing animals. These alterations in protein breakdown rates were associated with an inhibition of the ATP-ubiquitin-dependent proteolytic pathway (35% and 41% for 2.4 and 1.2 kb ubiquitin mRNA, and 57% for the C8 proteasome subunit, respectively). The cytokine did not modify the plasma levels of corticosterone and insulin in the tumour hosts. The present data give new insights into the mechanisms by which IL-15 exerts its preventive effect on muscle protein wasting and seem to warrant the implementation of experimental protocols involving the use of the cytokine in the treatment of pathological states characterized by TPH, particularly in skeletal muscle, such as in the present model of cancer cachexia. © 2000 Cancer Research Campaign


*British Journal of Cancer* (2000) **83**(4), 526–531
© 2000 Cancer Research Campaign
doi: 10.1054/ bjoc.2000.1299, available online at http://www.idealibrary.com on IDEAL®

### Interleukin-15 antagonizes muscle protein waste in tumour-bearing rats

**N Carbó[1], J López-Soriano[1], P Costelli[2], S Busquets[1], B Alvarez[1], FM Baccino[2,3], LS Quinn[4], FJ López-Soriano[1] and JM Argilés[1]**

[1]Departament de Bioquímica i Biologia Molecular, Facultat de Biologia, Universitat de Barcelona, Diagonal 645, 08071-Barcelona, Spain; [2]Dipartimento di Medicina ed Oncologia Sperimentale, Università di Torino, Torino, Italy; [3]Centro CNR di Immunogenetica ed Oncologia Sperimentale, Torino, Italy; [4]Geriatric Research, Education and Clinical Center, VA Puget Sound Health Care System, American Lake Division, Tacoma, Washington 98493, USA

**Summary** Tissue protein hypercatabolism (TPH) is an important feature in cancer cachexia, particularly with regard to the skeletal muscle. The Yoshida AH-130 rat ascites hepatoma is a model system for studying the mechanisms involved in the processes that lead to tissue depletion, since it induces in the host a rapid and progressive muscle wasting, primarily due to TPH. The present study was aimed at investigating if IL-15, which is known to favour muscle fibre hypertrophy, could antagonize the enhanced muscle protein breakdown in this cancer cachexia model. Indeed, IL-15 treatment partly inhibited skeletal muscle wasting in AH-130-bearing rats by decreasing (8-fold) protein degradative rates (as measured by $^{14}C$-bicarbonate pre-loading of muscle proteins) to values even lower than those observed in non-tumour-bearing animals. These alterations in protein breakdown rates were associated with an inhibition of the ATP-ubiquitin-dependent proteolytic pathway (35% and 41% for 2.4 and 1.2 kb ubiquitin mRNA, and 57% for the C8 proteasome subunit, respectively). The cytokine did not modify the plasma levels of corticosterone and insulin in the tumour hosts. The present data give new insights into the mechanisms by which IL-15 exerts its preventive effect on muscle protein wasting and seem to warrant the implementation of experimental protocols involving the use of the cytokine in the treatment of pathological states characterized by TPH, particularly in skeletal muscle, such as in the present model of cancer cachexia. © 2000 Cancer Research Campaign

**Keywords** IL-15; cancer cachexia; protein turnover; skeletal muscle; proteasome; ubiquitin

Malignant neoplasms frequently induce a progressive loss of lean body mass in the host, associated with marked alterations in endocrine and metabolic homeostasis. Skeletal muscle tissue, which accounts for almost half of the whole body protein mass, is severely affected in cancer cachexia (Lawson et al, 1982; Morrison, 1989; Tisdale, 1992). Muscle wasting in cachexia is associated with enhanced protein turnover rates (Kien and Camitta, 1983; 1987; Beck and Tisdale, 1989; Melville et al, 1990; Beck et al, 1991). In addition, cachexia tends to develop at rather late stages of neoplastic disease. Thus, preventing muscle wasting in cancer patients is of great potential clinical interest. Whether the negative protein balance results from altered rates of synthesis or breakdown or from changes on both sides of muscle protein turnover remains unclear (Lundholm et al, 1979; Emery et al, 1982; Pain et al, 1984; Tessitore et al, 1987*a*).

The Yoshida AH-130 rat ascites hepatoma is a suitable model system for studying the mechanisms involved in the establishment of cachexia. Its growth in the host causes rapid and progressive loss of body weight and tissue wasting, particularly in skeletal muscle. Acceleration of tissue protein breakdown accounts for most of the wasting in AH-130 bearers (Baccino et al, 1986; Tessitore et al, 1986; 1987*a*; 1993*a*). In particular, skeletal muscle hypercatabolism involves hyperactivation of the ATP-ubiquitin-dependent proteolytic system (Llovera et al, 1994). Detectable plasma levels of tumour necrosis factor-α (TNF) and perturbations in hormonal homeostasis (Tessitore et al, 1993*b*) may play an important role in forcing metabolic balance towards the catabolic side.

Interleukin-15 (IL-15) is a novel cytokine first identified in the supernatant of the monkey epithelial cell line CV-1/EBNA as a factor capable of stimulating T-cell activity (Grabstein et al, 1994), and enhancing their antitumour properties (Munger et al, 1995). Indeed, IL-15 induces T-cell proliferation (Bruton et al, 1994), enhances natural killer (NK) cell activity, upregulates production of NK-cell-derived cytokines, including γ-interferon (γ-IFN), granulocyte/macrophage-colony stimulating factor (GM-CSF) and TNF (Carson et al, 1994), and promotes NK cell survival (Carson et al, 1997). In addition, it stimulates proliferation and differentiation of B cells activated by anti-immunoglobulin M antibodies (Armitage et al, 1995) and may protect T cells and neutrophils from apoptosis (Akbar et al, 1996; Girard et al, 1996), being a potent inhibitor of apoptosis both in vitro and in vivo (Bulfone-Paus et al, 1997). Despite the lack of significant sequence homology, IL-15 shares many biological activities with IL-2 since its function are mediated through the β and γ chains of the IL-2 receptor (Giri et al, 1994). However, unlike IL-2, which is produced almost exclusively by activated T cells, the IL-15 gene is not expressed in these cells (Grabstein et al, 1994), but rather IL-15 mRNA has been detected in placenta, skeletal muscle, kidney, lung, heart (Grabstein et al, 1994) and intestinal epithelial cells (Reinecker et al, 1996).

Recently, Quinn et al (1995) have proposed an important role for IL-15 in skeletal muscle. Indeed, IL-15 can stimulate differentiated myocytes and muscle fibres to accumulate increased amounts of

*Received 8 December 1999*
*Revised 28 February 2000*
*Accepted 17 April 2000*

*Correspondence to:* JM Argilés contractile proteins (Quinn et al, 1995), suggesting that IL-15 may play a role in skeletal muscle fibre growth in vivo. Bearing all this in mind, it was the aim of the present investigation to assess the effects of IL-15 administration on the skeletal muscle protein turnover in rats bearing a cachexia-generating, fast-growing tumour.

### MATERIALS AND METHODS

### Animals, tumour inoculation and treatment

Male Wistar rats (Interfauna, Barcelona, Spain) weighing approximately 110–135 g were utilized. The animals were maintained on a regular light–dark cycle (light from 8 am to 8 pm) and had free access to food and water. The diet (Panlab, Barcelona, Spain) consisted of 54% carbohydrate, 17% protein and 5% fat (the residue was non-digestible material). Rats were divided into two groups, controls and tumour hosts. The latter received an intraperitoneal inoculum of $10^8$ AH-130 Yoshida ascites hepatoma cells obtained from exponential tumours (for details see Tessitore et al, 1987*a*). Both groups were further divided into treated and untreated, the former being administered a daily s.c. dose of IL-15 (100 μg $kg^{-1}$ b.w. dissolved in physiological saline solution), the latter a corresponding volume of vehicle.

On days 0, 4, and 7 after tumour transplantation, animals were weighed and anaesthesized with diethyl ether. The tumour was harvested from the peritoneal cavity, its volume and cellularity evaluated. Blood was collected from the abdominal aorta into heparinized tubes and centrifuged (3500 ***g***, 10 min, 4°C) to obtain plasma. Tissues were rapidly excised, weighed, and frozen in liquid nitrogen (Tessitore et al, 1987*a*; 1993*a*).

### Protein turnover

Protein turnover rates were determined by a method that, as previously discussed (Tessitore et al, 1987*a*, 1993*a*), offers the best compromise for monitoring protein synthesis and degradation simultaneously in the same animal (Garlick et al, 1975). Briefly, apparent rates of synthesis and degradation for proteins of the slow turnover pool were evaluated by measuring the decay in specific and total protein radioactivity in tibialis muscles after labeling in vivo, 24 h before tumour transplantation, with a single intraperitoneal dose of sodium $^{14}C$-bicarbonate (250 μCi $kg^{-1}$ b.w.). Four days after tumour inoculation, fractional rates of protein degradation ($k_d$), synthesis ($k_s$), and accumulation ($k_a$) were calculated as follows and expressed as % per day:

$$k_d = \ln(\text{total protein radioactivity})/t$$

$$k_s = \ln(\text{specific protein radioactivity})/t$$

$$k_a = \ln(\text{total protein})/t$$

Muscle protein extraction was carried out by using a trichloroacetic acid precipitation method, as previously described (Costelli et al, 1993). Tissue protein was determined by the method of Bradford (1976), using bovine serum albumin as working standard.

### RNA isolation and Northern blot analysis

Total RNA from tibialis muscle was extracted using the acid guanidinium isothiocyanate/phenol/chloroform method as described by Chomczynski and Sacchi (1987). RNA samples (20 μg) were denatured, subjected to 1.2% agarose gel electrophoresis and transferred to Hybond N membrane (Amersham). RNA was fixed to the membranes by Genelinker (Biorad).

The RNA in gel and filters was visualized with ethidium bromide and photographed by UV transillumination to ensure the integrity of RNA, to check the loading of equivalent amounts of RNA and to confirm proper transfer. RNA was trasferred in 20 × standard saline citrate (SSC: 0.15 M NaCl and 15 mM sodium citrate, pH 7.0). Hybridization was done at 65°C overnight in the hybridization buffer (0.25 M $Na_2HPO_4$, 7% SDS, 1 mM EDTA, 1% BSA, 10% dextran sulphate), and denatured labelled probes ($10^6$–$10^7$ cpm $ml^{-1}$) were added.

Radiolabelled probes were prepared by the random priming method (Boehringer-Mannheim). The ubiquitin probe used was a cDNA clone containing 12 base pairs of the second ubiquitin coding sequence plus a complete third and fourth ubiquitin coding sequence and 120 base pairs of the 3′-untranslated region of the chicken polyubiquitin gene UB1 (Bond and Schlesinger, 1985). The C8 proteasome subunit probe used was a cDNA clone containing 850 base pairs of the rat C8 proteasome gene (Tanaka et al, 1990). UV light-illuminated ethidium bromide staining of the 28S rRNA was used as a control of loading. Filters were exposed to Hyperfilm-MP films (Amersham) at –80°C for 2–4 days and quantified by scanning densitometry.

### Plasma hormones

Circulating corticosterone was evaluated by a rat radioimmunoassay (IDS, Boldon, England). Insulin was measured by radioimmunoassay by the method of Albano et al (1972), using rat insulin as working standard.

### Data presentation

Data are given as means ± SEM Student's *t*-test was used to calculate the significance of differences.

### Chemicals

All enzymes and coenzymes were obtained from Boehringer-Mannheim (Barcelona, Spain) or Sigma (St. Louis, MO, USA), sodium $^{14}C$-bicarbonate (53 mCi $mmol^{-1}$) from New England Nuclear (Boston, MA, USA). Recombinant human IL-15 was kindly provided by Immunex Corporation (Seattle, Washington, USA).

### RESULTS

The Yoshida AH-130 rat ascites hepatoma grew exponentially for 4–5 days then shifted into a stationary phase approximately 7 days after transplantation, as previously shown (Tessitore et al, 1987*b*). Tumour growth was not significantly affected by IL-15 (Table 1).

As shown in Table 1, the loss of body weight in tumour bearers became clear by day 7 and was less marked in the IL-15-treated tumour-bearing group. A reduction of white adipose tissue mass was detectable at day 4 in IL-15-treated controls; this tissue was also diminished in tumour bearers (both at days 4 and 7), yet not further affected to a significant degree by the cytokine treatment. Liver was hypotrophic 4 days after tumour inoculation and even more so by day 7, when a significant protective effect exerted by IL-15 on this tissue was observed.

© 2000 Cancer Research Campaign

**Table 1** Body and tissue weight and tumour growth in AH-130 hosts

| Time | Tumour | Treatment | Body weight (g) initial | Body weight (g) final | Liver (mg) | WAT (mg) | BAT (mg) | Total tumour cell number (× $10^6$) | Tumour volume (ml) |
|---|---|---|---|---|---|---|---|---|---|
| day 4 | no | none | 131 ± 4 | 166 ± 5 | 5850 ± 177 | 749 ± 32 | 149 ± 16 | | |
| | | IL-15 | 132 ± 5 | 166 ± 7 | 6031 ± 418 | 510 ± 82[d] | 111 ± 9 | | |
| | yes | none | 134 ± 5 | 150 ± 6 | 5005 ± 186[a] | 552 ± 34[b] | 122 ± 5 | 1978 ± 178 | 16 ± 2 |
| | | IL-15 | 135 ± 7 | 143 ± 8 | 4945 ± 219[a] | 614 ± 32[a] | 127 ± 14 | 2070 ± 178 | 16 ± 3 |
| day 7 | no | none | 111 ± 3 | 177 ± 4 | 7944 ± 123 | 838 ± 54 | 295 ± 10 | | |
| | | IL-15 | 111 ± 3 | 169 ± 4 | 7196 ± 56[e] | 539 ± 52[e] | 290 ± 17 | | |
| | yes | none | 115 ± 4 | 99 ± 4[c] | 4098 ± 105[c] | 269 ± 30[c] | 117 ± 6[c] | 4528 ± 156 | 54 ± 2 |
| | | IL-15 | 114 ± 3 | 111 ± 1[c,d] | 4769 ± 200[c,d] | 246 ± 21[c] | 125 ± 14[c] | 4895 ± 312 | 57 ± 3 |

Data are expressed as means ± SEM. Tissue weights are expressed as mg per 100 g of initial body weight. Final body weight excludes tumour weight. Significance of the differences (Student's *t*-test): a = $P < 0.05$, b = $P < 0.01$, c = $P < 0.001$ (vs non-tumour bearers); d = $P < 0.05$, e = $P < 0.01$ (vs. non-treated). $n$ = 4 and 6 for non-tumour and tumour bearers, respectively. WAT = white adipose tissue; BAT = brown adipose tissue

**Table 2** Muscle weight

| Time | Tumour | Treatment | GSN | Soleus | Tibialis | Heart |
|---|---|---|---|---|---|---|
| day 0 | no | none | 472 ± 5 | 37.4 ± 0.2 | 154 ± 2 | 382 ± 14 |
| day 4 | no | none | 569 ± 15 | 45.7 ± 1.1 | 179 ± 1 | 386 ± 9 |
| | no | IL-15 | 598 ± 14 | 44.3 ± 0.8 | 182 ± 5 | 386 ± 7 |
| | yes | none | 523 ± 7[a] | 40.5 ± 1.3[a] | 167 ± 4[a] | 355 ± 7[a] |
| | yes | IL-15 | 564 ± 9[c] | 43.9 ± 1.8 | 170 ± 8 | 358 ± 15 |
| day 7 | no | none | 683 ± 13 | 52.0 ± 0.3 | 215 ± 3 | 532 ± 14 |
| | no | IL-15 | 677 ± 13 | 55.6 ± 1.3 | 206 ± 5 | 524 ± 5 |
| | yes | none | 442 ± 13[b] | 37.5 ± 1.0[b] | 140 ± 4[b] | 360 ± 13[b] |
| | yes | IL-15 | 489 ± 7[b,c] | 42.1 ± 0.9[b,d] | 163 ± 3[b,d] | 387 ± 6[b] |

Data (means ± SEM) are expressed as mg per 100 g body weight. Statistical comparison of the data (Student's *t*-test): a = $P < 0.05$, b = $P < 0.001$ (vs non-tumour bearers); c = $P < 0.05$, d = $P < 0.01$ (vs non-treated). $n$ = 4 and 6 for non-tumour and tumour bearers respectively. GSN = gastrocnemius

**Table 3** Tibialis protein content

| Time | Tumour | Treatment | mg $g^{-1}$ tissue | mg protein per 100 g ibw |
|---|---|---|---|---|
| day 4 | no | none | 143 ± 4.4 | 25.5 ± 0.58 |
| | no | IL-15 | 141 ± 2.0 | 26.2 ± 0.35 |
| | yes | none | 144 ± 3.6 | 22.7 ± 2.05 |
| | yes | IL-15 | 146 ± 1.5 | 25.2 ± 0.97 |
| day 7 | no | none | 126 ± 1.4 | 27.0 ± 0.65 |
| | no | IL-15 | 129 ± 2.5 | 26.5 ± 0.56 |
| | yes | none | 108 ± 6.0[a] | 15.3 ± 1.18[b] |
| | yes | IL-15 | 119 ± 4.6 | 19.2 ± 0.79[b,c] |

Data are expressed as mean ± SEM. Statistical comparison of the data (Student's *t*-test): a = $P < 0.05$, b = $P < 0.001$ (vs non-tumour bearers); c = $P < 0.05$ (vs non-treated). $n$ = 4 and 6 for non-tumour and tumour bearers respectively. ibw = initial body weight.

Quite different was the pattern with regard to skeletal muscle. A decrease in wet weight (Table 2) and protein content (Table 3) was elicited by tumour growth, as previously reported (Tessitore et al, 1987*a*). Treatment with IL-15 partially prevented the protein waste in tibialis muscle (Table 3). This protective effect was also noticeable in terms of wet tissue weight for the skeletal muscles (Table 2). In agreement with previous observations (Carbó et al, 2000), IL-15 did not exert any trophic action (weight or protein increase) on muscles in non-tumour bearers (Tables 2 and 3).

Protein turnover in tibialis muscle 4 days after tumour inoculation was evaluated to determine if the protein-sparing action of IL-15 was exerted by changes in muscle protein degradation or synthesis, or both (Table 4). As previously reported (Tessitore et al, 1987*a*, 1993*a*), rates of tibialis muscle protein degradation were enhanced (48%) as a consequence of tumour growth, while muscle protein synthesis remained virtually unchanged, resulting in protein accumulation rates lower (27%) than in controls (Table 4). On treatment with IL-15, the elevation of protein breakdown rates was suppressed and protein accumulation rates returned to levels similar to those in controls (Table 4). No detectable effect was observed with regard to protein synthesis rates. In non-tumour bearers, IL-15 treatment resulted in a decrease in protein degradation

 *© 2000 Cancer Research Campaign*

**Table 4** Tibialis muscle protein turnover

| Time | Tumour | Treatment | $k_s$ | $k_d$ | $k_a$ |
|---|---|---|---|---|---|
| day 4 | no | none | 10.7 ± 0.86 | 2.77 ± 0.27 | 7.94 ± 0.63 |
| | no | IL-15 | 9.39 ± 1.54 | 0.82 ± 0.20[b] | 8.22 ± 0.93 |
| | yes | none | 10.6 ± 1.54 | 4.11 ± 0.82 | 5.76 ± 1.23 |
| | yes | IL-15 | 8.80 ± 0.75 | 0.49 ± 0.49[a,c] | 8.07 ± 0.98 |

For further details see Materials and methods. Fractional rates of protein synthesis ($k_s$), degradation ($k_d$) and accumulation ($k_a$) are expressed as % per day ($n$ = 5 for each time-point) and were calculated over the time interval 0–4 days. Significance of the differences (Student's *t*-test): a = $P < 0.01$, b = $P < 0.001$ (vs controls); c = $P < 0.01$ (vs AH-130)

(70%) and synthesis (12%) rates, which resulted in no changes in protein accumulation (Table 4).

As previously shown (Llovera et al, 1994), the accelerated muscle protein breakdown in the AH-130 hosts is associated with activation of the ATP-ubiquitin-dependent proteolytic system. Two polyubiquitin mRNA species (2.4 kb and 1.2 kb) were found in tibialis muscle (Figure 1). 7-day tumour-bearing animals showed an increased expression of the polyubiquitin genes compared with the corresponding control animals: over 3-fold for both ubiquitin transcripts. The expression of the C8 proteasome subunit was also increased as a result of tumour burden (Figure 1). Control animals receiving IL-15 also showed significant decreases in the expression of these genes, in agreement with the decreased protein degradation associated with IL-15 treatment (Table 4). In addition, when the tumour-bearing animals received IL-15, the activation of this proteolytic system was also suppressed (Figure 1).

Corticosterone was elevated (day 7) and insulin decreased (days 4 and 7) in the blood plasma of AH-130 hosts at day 7, as previously observed (Tessitore et al, 1993*b*). When these animals were administered IL-15, neither insulin nor corticosterone levels were significantly affected, suggesting that the effects of the cytokine were not mediated through these hormones. In non-tumour bearers, the treatment did not modify insulin concentrations (Table 5).

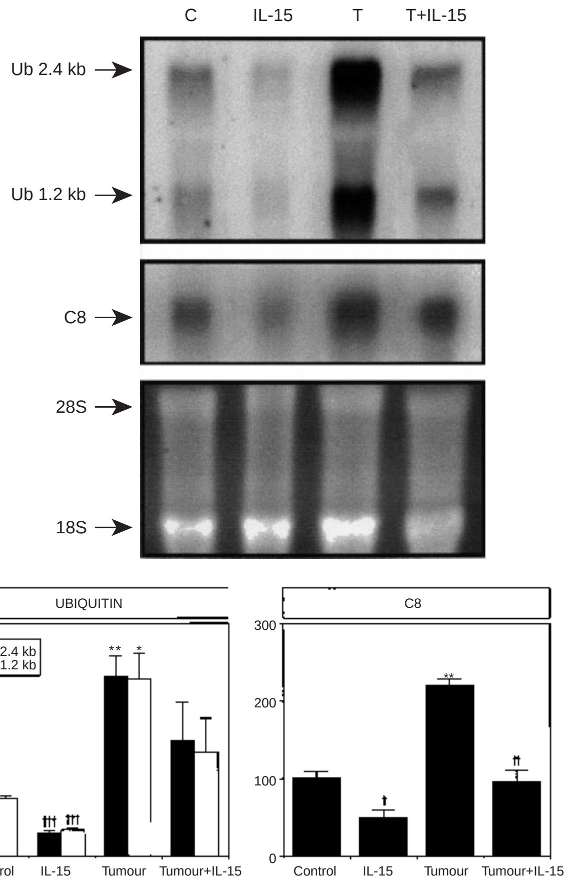

**Figure 1** Northern blots of tibialis muscle extracts from tumour-bearing rats. Expression of ubiquitin and C8 proteasome subunit mRNAs in tibialis muscles from control (C), control treated with IL-15 (IL-15), 7-day tumour-bearing (T) and 7-day tumour-bearing treated with IL-15 (T+IL-15). mRNA was detected after hybridization with a cDNA probe containing a region of the chicken polyubiquitin gene UB1 or the C8 gene. Autoradiographs were subjected to scanning densitometry. Ethidium bromide (EtBr) was used for total RNA quantitation in order to correct the results

### DISCUSSION

Muscle protein wasting is a prominent feature in cancer cachexia and is primarily ascribed to enhanced tissue protein catabolism (Kien and Camitta, 1983; 1987; Beck and Tisdale, 1989; Melville et al, 1990; Beck et al, 1991; Tessitore et al, 1993*a*). Most therapeutic approaches to cancer cachexia have been designed on the assumption that tissue wasting merely results from undernutrition or tumour–host competition, yet parenteral nutrition or overfeeding have proven to be only marginally or, at best, temporarily effective (Moley et al, 1988; Popp et al, 1988; Shaw and Wolfe, 1988). Moreover, in some cases the tumour itself apparently took more advantage of such a treatment than the patient (Tayek et al, 1986; Popp et al, 1988).

The rat tumour model used in the present study quickly causes progressive body weight loss and tissue protein wasting. The latter is associated with tissue protein hypercatabolism (TPH) (Tessitore et al, 1987*a*; 1987*b*; 1993*a*) which is probably mediated by production of cytokines such as TNF (García-Martínez et al, 1993; Llovera et al, 1993*a*, 1993*b*) and alterations in hormonal homeostasis (Tessitore et al, 1993a). The beneficial effects of treatments with anti-TNF antibodies (Costelli et al, 1993) or with drugs interfering with the development of TPH (Tessitore et al, 1994) have been previously reported. The present observations show that treatment of the AH-130 hosts with IL-15 inhibits some of the protein loss in the tibialis muscle by reverting the increase in protein degradation. This is consistent with previous reports indicating that IL-15 may act as an anabolic cytokine for skeletal muscle (Quinn et al, 1995). IL-15 activates NK cells (Carson et al, 1994; 1997), which have anti-tumour properties; however, it has to be pointed out that IL-15 had no effect on tumour growth in our model. Thus, the effects of IL-15 on tumour-induced wasting could not be related to the anti-tumour action of this cytokine.

The precise mechanisms by which intracellular proteins are degraded are largely unknown, although it is accepted that proteolysis may occur inside and outside the lysosomes. Lysosomal proteases, in particular cathepsins, are not the major mediators of myofibrillar protein degradation in rat skeletal muscle (Lowell et al, 1986; Tessitore et al, 1994). The ATP-ubiquitin-dependent proteolytic system is postulated to account for the turnover of short-lived proteins (Ciechanover et al, 1984) or for abnormal proteins formed during stress such as heat-shock (Bond et al, 1988). However, it has been suggested that the activity of this

© 2000 Cancer Research Campaign

**Table 5** Plasma levels of insulin and corticosterone

| Time | Tumor | Treatment | Corticosterone (ng ml$^{-1}$) | Insulin (ng ml$^{-1}$) |
|---|---|---|---|---|
| days 4–7 | no | none | 130 ± 15 | 2.31 ± 0.66 |
| day 4 | no | IL-15 | 77 ± 7[c] | 1.78 ± 0.63 |
| | yes | none | 234 ± 95 | 1.08 ± 0.32[a] |
| | yes | IL-15 | 120 ± 22 | 0.71 ± 0.05[a] |
| day 7 | no | IL-15 | 203 ± 22[c] | 2.78 ± 0.66 |
| | yes | none | 824 ± 178[b] | 1.57 ± 0.05[a] |
| | yes | IL-15 | 557 ± 107[b] | 1.67 ± 0.17[a] |

Data are means ± S.E.M. Significance of the differences (Student's t test): a = $P < 0.05$, b = $P < 0.001$ (vs control days 4–7); c = $P < 0.05$ (vs non-treated). $n$ = 4 and 6 for non- tumour and tumour bearers respectively system can also be related to the turnover of long-lived proteins such as those found in skeletal muscle (Hilenski et al, 1992). Recently, this proteolytic system has been involved in the perturbations of protein metabolism in skeletal muscle in many pathological conditions (see Argilés and López-Soriano, 1996 for review). Previous studies from our laboratory have shown that the lysosomal pathway is only marginally involved in the development of muscle protein hypercatabolism in the AH-130 hosts (Llovera et al, 1994; 1995; Tessitore et al, 1994), while an important activation of the ATP-dependent proteolysis seems to be the major mechanism (Llovera et al, 1994; Costelli et al, 1995). It is very interesting to observe that the preventive effect exerted by IL-15 on the acceleration of muscle protein breakdown is associated with a normalization of the hyperexpression of both the C8 proteasome subunit and the ubiquitin mRNAs. Moreover, the present observation that IL-15 administration to control rats reduces ubiquitin and C8 mRNAs expression below the basal values should be stressed. To the best of our knowledge, this is the first report showing that the effect of IL-15 on muscle protein turnover may be due to downregulation of the ATP-ubiquitin-dependent proteolytic system.

The action of the cytokine on muscle protein metabolism in skeletal muscle might involve hormonal modulations. However, as far as insulin and corticosterone are concerned, the present study suggests that this is not the case for the AH-130 hosts, since the altered plasma level of neither hormone was corrected by the treatment.

Another important action of IL-15 is to reduce the body fat content by inhibiting lipogenesis (Carbó et al, 2000); tissue-specific modulations in lipoprotein lipase activity could provide an additional mechanism for promoting a decreased lipid accretion in adipose tissue. The present cachexia model is itself characterized by extensive lipid mobilization (Dessì et al, 1992; Carbó et al, 1994), associated with decreased activity of tissue lipoprotein lipase (Carbó et al, 1994). The lack of effect of IL-15 on adipose tissue in AH-130 hosts may thus reflect the fact that lipid mobilization was already occurring in these animals and could not easily be stimulated further. When the cytokine treatment was applied to control rats, however, adipose tissue mass decreased with respect to untreated controls (by 34%), in agreement with previous reports (Carbó et al, 2000).

In conclusion, the present results indicate that IL-15 exerted a selective, protective action on skeletal muscle by antagonizing both the enhanced protein degradation and, to some extent the resulting protein loss, which characterize cachexia in AH-130 tumour-bearing rats. These observations suggest that the cytokine could be a convenient therapeutic tool in pathological states where muscle protein hypercatabolism is a critical feature, such as cancer cachexia or other wasting diseases.

### ACKNOWLEDGEMENTS

Work supported by grants from the Fondo de Investigaciones Sanitarias de la Seguridad Social (97/2059) of the Spanish Health Ministry, the Dirección General de Investigación Científica y Técnica (PB94-0938 and HI-1996–0049) from the Spanish Ministry of Education and Science, the Ministero dell'Università e della Ricerca Scientifica e Tecnologica (60% and 40% funds), Roma, the Consiglio Nazionale delle Ricerche (Special Project ACRO), Roma, the Associazione Italiana per la Ricerca sul Cancro, Milano. The authors would like to thank Immunex Corporation (Seattle, WA, USA), which kindly provided IL-15, and Dr MJ Schlesinger and Dr K Tanaka for providing the ubiquitin-specific and proteasome C8 probes, respectively.

### REFERENCES

Akbar AN, Borthwick NJ, Wickremashinge RG, Panayoitidis P, Pilling D, Bofill M, Krajewski S, Reed JC and Salmon M (1996) IL-2 receptor γ-chain signalling cytokines regulate activated T-cell apoptosis in response to growth factor withdrawal: selective induction of anti-apoptotic (bcl-2, bcl-$X_L$) but not proapoptotic (bax, bcl-$X_S$) gene expression. *Eur J Immunol* **26:** 294–299

Albano JDM, Ekins RP, Maritz G and Turner RC (1972) A sensitive and precise radioimmunoassay of serum insulin. *Acta Endocrinol* **70:** 487–509

Argilés JM and López-Soriano FJ (1996) The ubiquitin-dependent proteolytic pathway in skeletal muscle: its role in pathological states. *Trends Pharmacol Sci* **17:** 223–226

Armitage RJ, Macduff BM, Eisenman J, Paxton R and Grabstein KH (1995) IL-15 has stimulatory activity for the induction of B cell proliferation and differentiation. *J Immunol* **154:** 483–490

Baccino FM, Tessitore L, Bonelli G and Isidoro C (1986) Protein turnover states of tumour cells and host tissues in an experimental model. *Biomed Biochim Acta* **45:** 1585–1590

Beck SA and Tisdale MJ (1989) Nitrogen excretion in cancer cachexia and its modification by a high fat diet in mice. *Cancer Res* **49:** 3800–3804

Beck SA, Smith KL and Tisdale MJ (1991) Anticachectic and antitumour effect of eicosapentaenoic acid and its effect on protein turnover. *Cancer Res* **51:** 6089–6093

Bond U and Schlesinger MJ (1985) Ubiquitin is a heat-shock protein in chicken-embryo fibroblasts. *Mol Cell Biol* **5:** 949–956

Bond U, Agell N, Haas AL, Redman K and Schlesinger MJ (1988) Ubiquitin in stressed chicken embryo fibroblasts. *J Biol Chem* **263:** 2384–2388

Bradford MM (1976) A rapid and sensitive method for the quantification of microgram quantities of protein utilizing the principle protein-dye binding. *Anal Biochem* **72:** 248–254

Bruton JD, Bamford RN, Peters C, Grant AJ, Kurys G, Goldman CK, Brennan J, Roessler E and Waldmann TA (1994) A lymphokine, provisionally designated interleukin T and produced by a human adult T cell leukemia line, stimulates T cell proliferation and the induction of lymphokine-activated killer cells. *Proc Natl Acad Sci USA* **91:** 4935–4939

Bulfone-Paus S, Ungureanu D, Pohl T, Lindner G, Paus R, Ruckert R, Krause H and Kunzendorf U (1997) Interleukin-15 protects from lethal apoptosis in vivo. *Nature Med* **3:** 1124–1128

Carbó N, Costelli P, Tessitore L, Bagby GJ, López-Soriano FJ, Baccino FM and Argilés JM (1994) Anti-TNF treatment interferes with changes in lipid metabolism in a tumour cachexia model. *Clin Sci* **87:** 349–355

Carbó N, López-Soriano J, Costelli P, Alvarez B, Busquets S, Baccino FM, Quinn LS, López-Soriano FJ and Argilés JM (2000) Interleukin-15 mediates reciprocal regulation of adipose tissue and muscle mass: a potential role in body weight control. Submitted to publication

Carson WE, Giri JG, Lindemann MJ, Linett ML, Ahdieh M, Paxton R, Anderson D, Eisenman J, Grabstein K and Caligiuri MA (1994) Interleukin (IL) 15 is a novel cytokine that activates human natural killer cells via components of the IL-2 receptor. *J Exp Med* **180:** 1395–1403

 *© 2000 Cancer Research Campaign*

Carson WE, Fehniger TA, Haldar S, Eckhert K, Lindemann MJ, Lai CF, Croce CM, Baumann H and Caligiuri MA (1997) A potential role for interleukin-15 in the regulation of human natural killer cell survival. *J Clin Invest* **99:** 937–943

Chomczynski P and Sacchi N (1987) Single-step method of RNA isolation by acid guanidinium thiocyanate phenol chloroform extraction. *Anal Biochem* **162:** 156–159

Ciechanover A, Finley D and Varshavsky A (1984) Ubiquitin dependence of selective protein degradation demonstrated in the mammalian cell cycle mutant ts85. *Cell* **37:** 57–66

Costelli P, Carbó N, Tessitore L, Bagby GJ, López-Soriano FJ, Argilés JM and Baccino FM (1993) Tumour necrosis factor-α mediates changes in protein turnover in a rat cancer cachexia model. *J Clin Invest* **92:** 2783–2789

Costelli P, García-Martínez C, Llovera M, Carbó N, López-Soriano FJ, Agell N, Tessitore L, Baccino FM and Argilés JM (1995) Muscle protein waste in tumor-bearing rats is effectively antagonized by a $\beta_2$-adrenergic agonist (clenbuterol). *J Clin Invest* **95:** 2367–2372

Dessì S, Batetta B, Anchisi C, Pani P, Costelli P, Tessitore L and Baccino FM (1992) Cholesterol metabolism during the growth of a rat ascites hepatoma (Yoshida AH-130). *Br J Cancer* **66:** 787–793

Emery PW, Neville AM, Edwards RHT and Rennie MJ (1982) Increased myofibrillar degradation and decreased protein synthesis in tumor-bearing mice. *Eur J Clin Invest* **12:** 10

García-Martínez C, López-Soriano FJ and Argilés JM (1993) Acute treatment with tumour necrosis factor-α induces changes in protein metabolism in rat skeletal muscle. *Mol Cell Biochem* **125:** 11–18

Garlick PJ, Millward DJ, James WPT and Waterlow JC (1975) The effect of protein deprivation and starvation on the rate of protein synthesis in tissues of the rat. *Biochim Biophys Acta* **414:** 71–84

Girard D, Paquet ME, Paquin R and Beaulieu AD (1996) Differential effects of interleukin-15 (IL-15) and IL-2 on human neutrophils: modulation of phagocytosis, cytoskeleton rearrangement, gene expression, and apoptosis by IL-15. *Blood* **88:** 3176–3184

Giri JG, Ahdieh M, Eisenman J, Shanebeck K, Grabstein K, Kumaki S, Namen A, Park LS, Cosman D and Anderson D (1994) Utilization of the β and γ chain of the IL-2 receptor by the novel cytokine IL-15. *EMBO J* **13:** 2822–2830

Grabstein KH, Eisenman J, Shanebeck K, Rauch C, Srinivasan S, Fung V, Beers C, Richardson J, Schoenborn MA, Ahdieh M, Johnson L, Alderson MR, Watson JD, Anderson DM and Giri JG (1994) Cloning of a T cell growth factor that interacts with the β chain of the interleukin-2 receptor. *Science* **264:** 965–968

Hilenski LL, Terracio L, Haas AL and Borg TK (1992) Immunolocalization of ubiquitin conjugates at Z-bands and intercalated discs of rat cardiomyocytes in vitro and in vivo. *J Histochem Cytochem* **40:** 1037–1042

Kien CL and Camitta BM (1983) Increased whole-body protein turnover in children with newly diagnosed leukemia or lymphoma. *Cancer Res* **43:** 5592–5596

Kien CL and Camitta BM (1987) Close association of accelerated rates of whole body protein turnover (synthesis and breakdown) and energy expenditure in children with newly diagnosed acute lymphocytic leukaemia. *J Parent Enteral Nutr* **11:** 129–134

Lawson DH, Richmond A, Nixon DW and Rudman D (1982) Metabolic approaches to cancer cachexia. *Annu Rev Nutr* **2:** 277–301

Llovera M, López-Soriano FJ and Argilés JM (1993*a*) Effects of tumour necrosis factor-α on muscle protein turnover in vivo in female rats. *J Natl Cancer Inst* **85:** 1334–1339

Llovera M, López-Soriano FJ and Argilés JM (1993*b*) Chronic tumour necrosis factor-α treatment modifies protein turnover in rat tissues. *Biochem Mol Biol Int* **30:** 29–36

Llovera M, García-Martínez C, Agell N, Marzábal M, López-Soriano FJ and Argilés JM (1994) Ubiquitin gene expression is increased in skeletal muscle of tumour-bearing rats. *FEBS Lett* **338:** 311–318

Llovera M, Garcia-Martínez C, Agell N, López-Soriano FJ and Argilés JM (1995) Muscle wasting associated with cancer cachexia is linked to an important activation of the ATP-dependent ubiquitin-mediated proteolysis. *Int J Cancer* **61:** 138–141

Lowell BB, Ruderman NB and Goodman MN (1986) Evidence that lysosomes are not involved in the degradation of myofibrillar proteins in rat skeletal muscle. *Biochem J* **234:** 237–240

Lundholm K, Ekman D, Edstrom S, Karlberg I, Jagenberg R and Scherstén T (1979) Protein synthesis in liver tissue under the influence of a methyl-cholanthrene-induced sarcoma in mice. *Cancer Res* **39:** 4657–4661

Melville S, McNurlan MA, Graham Calder A and Garlick PJ (1990) Increased protein turnover despite normal energy metabolism and responses to feeding in patients with lung cancer. *Cancer Res* **50:** 1125–1131

Moley JF, Morrison SD, Gorschboth CM and Norton JA (1988) Body composition changes in rats with experimental cancer cachexia: improvement with exogenous insulin. *Cancer Res* **48:** 2784–2787

Morrison SD (1989) Cancer cachexia. In *Influence of Tumor Development on the Host* (Liotta AL, ed), pp. 176–213. Kluwer Academic Publ., The Netherlands.

Munger W, DeJoy SQ, Jeyaseelan R Sr, Torley LW, Grabstein KH, Eisenman J, Paxton R, Cox T, Wick MM and Kerwar SS (1995) Studies evaluating the antitumor activity and toxicity of interleukin-15, a new T cell growth factor: comparison with interleukin-2. *Cell Immunol* **165:** 289–293

Pain VM, Randall DP and Garlick PJ (1984) Protein synthesis in liver and skeletal muscle of mice bearing an ascites tumor. *Cancer Res* **44:** 1054–1057

Popp MB, Wagner SC, Enrione EB and Brito OJ (1988) Host and tumor responses to varying rates of nitrogen infusion in the tumor-bearing rat. *Ann Surg* **207:** 80–89

Quinn LS, Haugk KL and Grabstein KH (1995) Interleukin-15: a novel anabolic cytokine for skeletal muscle. *Endocrinology* **136:** 3669–3672

Reinecker HC, MacDermott RP, Mirau S, Dignass A and Podolsky DK (1996) Intestinal epithelial cells both express and respond to interleukin-15. *Gastroenterology* **111:** 1706–1713

Shaw JH and Wolfe RR (1988) Whole-body protein kinetics in patients with early and advanced gastrointestinal cancer: the early response to glucose infusion and total parenteral nutrition. *Surgery* **103:** 148–155

Tanaka K, Kanamaya H, Tamura T, Lee DH, Kumatori A, Fujiwara T, Ichihara A, Tokunaga F, Aruga R and Iwanaga S (1990) cDNA cloning and sequencing of component C8 of proteasomes from rat hepatoma cells. *Biochem Biophys Res Commun* **171:** 676–683

Tayek JA, Istfan NW, Jones CT, Hamawy KJ, Bistrian BR and Blackburn GL (1986) Influence of the Walker 256 carcinosarcoma on muscle, tumor, and whole-body protein synthesis and growth rate in the cancer-bearing rat. *Cancer Res* **46:** 5649–5654

Tessitore L, Bonelli G, Isidoro C, Kazakova OV and Baccino FM (1986) Comparative studies of protein turnover regulations in tumor cells and host tissues: development and analysis of an experimental model. *Toxicol Pathol* **14:** 451–456

Tessitore L, Bonelli G and Baccino FM (1987*a*) Early development of protein metabolic perturbations in the liver and skeletal muscle of tumour-bearing rats. *Biochem J* **241:** 153–159

Tessitore L, Bonelli G, Cecchini G, Amenta JS and Baccino FM (1987*b*) Regulation of protein turnover versus growth state: ascites hepatoma as a model for studies both in the animal and in vitro. *Arch Biochem Biophys* **255:** 372–384

Tessitore L, Costelli P, Bonetti G and Baccino FM (1993*a*) Cancer cachexia, malnutrition, and tissue protein turnover in experimental animals. *Arch Biochem Biophys* **306:** 52–58

Tessitore L, Costelli P and Baccino FM (1993*b*) Humoral mediation for cancer cachexia in tumour-bearing rats. *Br J Cancer* **67:** 15–23

Tessitore L, Costelli P and Baccino FM (1994) Pharmacological interference with tissue hypercatabolism in tumour-bearing rats. *Biochem J* **299:** 71–78

Tisdale MJ (1992) Cancer cachexia. *Br J Cancer* **63:** 337–342

© 2000 Cancer Research Campaign 
